# Home-based system for physical activity monitoring in patients with multiple sclerosis (Pilot study)

**DOI:** 10.1186/1475-925X-13-10

**Published:** 2014-02-06

**Authors:** Layal Shammas, Tom Zentek, Birte von Haaren, Stefan Schlesinger, Stefan Hey, Asarnusch Rashid

**Affiliations:** 1 FZI Forschungszentrum Informatik, Karlsruhe, Germany; 2 hiper campus, Karlsruhe Institute of Technology (KIT) Karlsruhe Germany, Karlsruhe, Germany; 3 Rhön Klinikum Bad Neustadt/Saale, Neustadt an der Saale, Germany

**Keywords:** Daily physical activity, Home-Based activity monitoring, Physical activity fluctuation, 3D acceleration sensor, Physical activity level, Gait speed, Step count, Expanded disability status scale (EDSS), Multiple sclerosis

## Abstract

**Background:**

Limitations in physical activity are considered as a key problem in patients with multiple sclerosis (PwMS). Contemporary methods to assess the level of physical activity in PwMS are regular clinical observation. However, these methods either rely on high recall and accurate reporting from the patients (e.g. self-report questionnaires), or they are conducted during a particular clinical assessment with predefined activities. Therefore, the main aim of this pilot study was to develop an objective method to gather information about the real type and intensity of daily activities performed by PwMS in every-day living situations using an accelerometer. Furthermore, the accelerometer-derived measures are investigated regarding their potential for discriminating between different MS groups.

**Methods:**

Eleven PwMS that were able to walk independently (EDSS ≤ 5) were divided into two groups: mild disability (EDSS 1–2.5; n = 6) and moderate disability (EDSS 3 –5; n = 5). Participants made use of an activity monitor device attached to their waist during their normal daily activities over 4 measurements. Activity parameters were assessed and compared for the time of each participant’s first measurement and follow-up measurement. Furthermore, differences between both subgroups, and the correlation of activity parameters with the clinical neurological variable (EDSS) were investigated.

**Results:**

Participants showed significant decline in step count (p = 0.008), maximum walking speed (p = 0.02) and physical activity intensity (p = 0.03) throughout the study period. Compared to the mild subgroup, moderate affected participant accumulated less number of steps (G1: 9214.33 ± 2439.11, G2: 5018.13 ± 2416.96; p < 0.005) and were slower (G1: 1.48 ± 0.19, G2: 1.12 ± 0.44; p = 0.03). Additionally, the EDSS correlated negatively with mean walking speed (r = - 0.71, p = 0.01) and steps count (r = - 0.54, p = 0.08).

**Conclusions:**

In this study, we used a portable activity monitoring sensor to gather information about everyday physical activity in PwMS at home. We showed that objective measurements using simple 3D accelerometers can track daily physical activity fluctuation. Furthermore, they track disability changes better than clinical measures. Thus, they can help to develop activity based treatments for PwMS.

## Background

Multiple sclerosis (MS) is an autoimmune disease of the central nervous system in which patients often exhibit decreasing physical activity and reduced independence [1]. Such severe inactivity is associated with worsening of disability in PwMS [2]. Commonly used clinical measures of physical function in individuals with multiple sclerosis - such as a 6-Minute Walk test (6MWT) [3] and a Timed 25-Foot Walk Test (T25FW) [4] - do not reflect the patients’ situation during daily life. Therefore, many researchers have increasingly directed their attention towards understanding activity behaviour in PwMS within their customary environment [5]. Different methods have been used for this purpose, including subjective approaches like self-report and activity diaries as well as more objective methods using devices such as pedometers, gyroscopes and accelerometers [6].

However, nearly half of all PwMS develop cognitive dysfunction, like deficits in recent memory, which might influence the accuracy of the physical activity recall [7,8]. Furthermore, different studies have shown that the self-reporting methods are prone to error due to memory failure and other kind of misreporting [9,10]. In contrast to these subjective methods, objective devices are unobtrusive and can provide precise insight into the physical activity behaviour in PwMS [11]. Recent advances in technology have promoted the development of objective methods to allow continuous monitoring of the daily physical activity of multiple populations, such as stroke survivors, Parkinson’s disease (PD) patients and PwMS [8,12,13]. For example, White et al. examined the reliability of functional activity measured by an activity monitor in individuals with Parkinson’s disease in their customary home and community [14]. Salarian et al. examined a method for ambulatory monitoring of physical activity of PD patients during their daily activity and analysed a pattern of sit-to-stand transitions by placing three inertial sensors, two gyroscopes and one accelerometer, on different parts of the body [15]. Busse et al. used a Step Watch to investigate the accuracy and reliability of ambulatory monitoring in PwMS [16]. The study of Motl et al. involved an evaluation of the accuracy of pedometers attached to the hip of PwMS under controlled laboratory conditions [17]. A home-based 24-hour ambulatory monitoring system using a tri-axial accelerometer has been used in the study of Rietberg et al. to investigate the feasibility and reproducibility of the ambulatory monitoring method to measure physical activity in PwMS [18]. Although pedometers are inexpensive and a commonly used tool to measure physical activity, they have a major drawback: they cannot reflect the intensity of the patients’ movements, i.e. the change in physical activity level, like increases in moderate or vigorous physical activity or reduction in sedentary time [19]. Furthermore, they might suffer from inaccuracy during self-selected and slow walking speeds in comparison with accelerometer [20]. Using gyroscopes will decrease the autonomy of the monitoring system due to their high power consumption [21,22]. Therefore, accelerometers are the best choice for the purpose of this study, as they provide more precise information regarding physical activity [23] and their battery life is assured for several days. Moreover, they are relatively unobtrusive so that patients are unrestricted. Different studies have investigated the reliability of acceleration sensors under free life controlled conditions [24]. Several research groups aimed independently to understand the free-living physical activity habitual of PwMS. For example, the study of Molt et al. [25] assessed the physical activity behaviour of patients with multiple sclerosis using accelerometer, pedometer and questionnaire during a 7-day period. Klassen et al. [26] explored the relationships between two measures of free-living physical activity (tri-axial accelerometer and activity diary). Molt et al. [27] examined the relation between disability progression in PwMS and their physical activity behaviour under free-living conditions. Molt et al. [28] investigated in their study the association between physical activity measured as activity counts and disability. There were a significant and medium size correlation between EDSS and daily activity counts. Difficulty in walking has been considered as the most visible sign of functional impairment in PwMS and it can decline in early stages of the disease [29]. Therefore, recent researches aimed to understand the correlation between disability and accelerometer output and walking impairment. The study of Sosnoff et al. [30] showed that walking speed has a strong correlation with disease severity in PwMS. Weikert et al. and Sosnoff et al. [31,32] measured the correlation between daily movement counts and real-life walking impairments in PwMS. A strong correlation between accelerometer metrics and walking impairment was reported in these studies. However, these previous researches measured walking impairments using either self-reported measures (e.g. MSWS-12) [33] or a brief assessment tool under non-familiar conditions (e.g. 6MWS), which provide limited information about walking in real-life environments. Another drawback is that the researchers used activity counts to measure the intensity of the physical activity. This method depends on one single regression model, which is not applicable on all types of activities. Moreover, it is in lack of information on duration of physical activity, which has an influence on the energy expenditure. Another drawback is that some of these previous studies used a uniaxial accelerometer to detect the movement in the vertical direction. These kinds of accelerometers are not able to detect the non-vertical body movements while walking [34].

The main purpose of our research is to capture the slight changes in the free-living activity behaviours in PwMS objectively to be able to understand the utility of these changes in the therapeutic practice. Moreover, we aimed to develop and provide a Home-based system to help doctors monitor the changes in the ambulatory physical activity of PwMS objectively. In contrast to other studies [35] we used an activity-dependent model to estimate the energy expenditure (EE) and to determine the intensity of the physical activity. This method uses more information from the acceleration signal and applies different estimation models of EE [36]. Moreover, we monitored the change in physical activity and walking impairment parameters over one year in 4 measurements phases with 3 months intervals. In contrast to the recent published study [37] which monitored the physical activity in PwMS over 2.5 years, in our study the measurements frequency was more often. This frequency is important to be able to capture the minimal changes in the physical activity behaviour even in absence of clinical signs. On the other hand, we used a tri-axial accelerometer in order to measure the changes in physical activity intensity, daily number of steps and walking speed objectively. Moreover, we analysed the correlation between these changes and disease progress in mild and moderate ambulatory limitation subgroups. Therefore, we conducted a pilot study in an ambulant setting in Germany.

We hypothesised that: 1) PwMS will show significant reduction in ambulatory activity over the follow-up period (4 phases), 2) physical activity parameters derived from the acceleration signal will differ significantly between two patients’ subgroups, 3) parameters of physical activity collected in the patients everyday environment associate significantly with clinical measures of disease severity, 4) objective measures of ambulatory activity parameters will be more responsive to the progressive changes of the disability in PwMS than the clinical measures.

## Methods

### Measurements system

**Figure 1:**
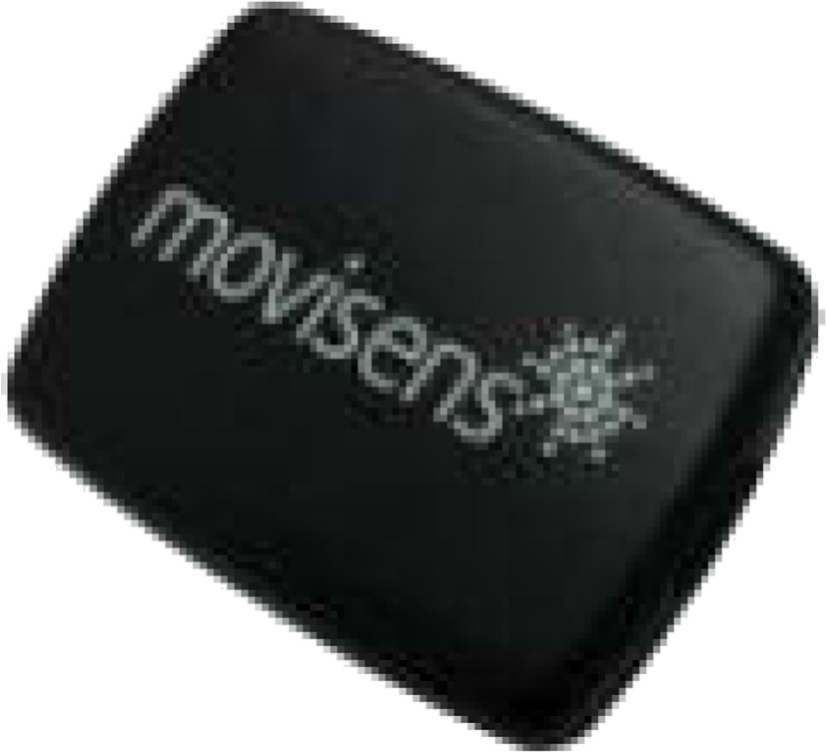
**Monitor device.** *Move II* a tri-axial accelerometer.

We used the *move II* activity sensor (movisens GmbH, Karlsruhe, Germany) to collect the activity data (see Figure 1). *Move II* consist of a 3-axial acceleration sensor (adxl345, Analog Device) with a range of ±8 g, a sample rate of 128 Hz and a resolution of 12 bit, and an air pressure sensor (BMP085, Bosch GmbH) with a sample rate of 8 Hz and a resolution of 0.03 hPa. The sensor weights 32 g, has the dimensions of 3 cm × 5 cm × 2 cm, and can be carried at different body positions (hip, wrist or chest). The acceleration signal can be recorded up to 30 days and saved on a 2 GB micro SD card. The recorded raw data can be transferred to a computer by USB 2.0 interface. The validity of *move II* was investigated in different studies. One study validated the accuracy of this accelerometer system in capturing the steps during controlled walking against another accelerometer model [38]. Other studies investigated the accuracy and validity of the *move II* in classifying physical activity and estimating energy expenditure [39,40]. *Move II* sensors are adapted for long-term monitoring of every-day living physical activities - including standing, sitting, lying, walking or running - in the course of daily life [39].

### Participants

Over a period of one year, 11 PwMS (females = 7, males = 4; age: 41 ± 9.3 year; height: 170 ± 8.12 cm; weight: 72 ± 16.77 kg; disease duration: 12.18 ± 10.67) were recruited in the Neurologische Klinik Bad Neustadt, a hospital for neurologic acute and rehabilitation medicine in a rural area in Northern Bavaria, Germany. Participants had to meet the following inclusion and exclusion criteria: a) definite diagnosis of MS [6]; b) EDSS score below 5 (3.6 ± 1.66), which indicates the ability to walk at least 200 m without assistive devices [41]; c) a completed and signed an informed consent. Eight patients had relapsing-remitting multiple sclerosis, one patient had primary progressive multiple sclerosis and two patients had secondary progressive multiple sclerosis.

### Study design

The procedure of this study was approved by the ethics committee of the Bavarian Medical Association, Germany.

**Figure 2:**
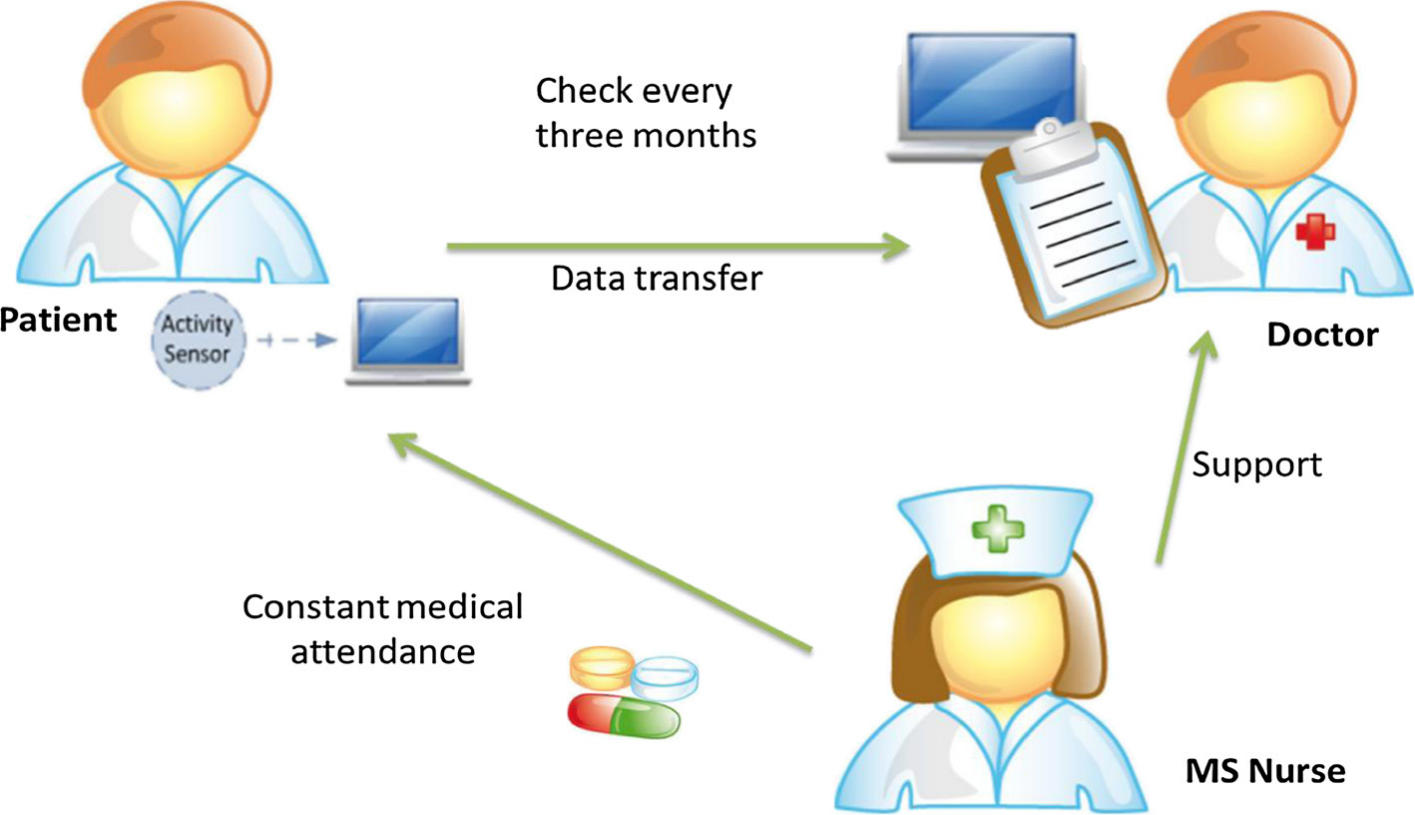
Study design.

The study lasted one year and consisted of clinical measures and ambulatory activity measures. These measures were collected four times (four phases), each lasting 10 days with an interval of three months between each phase. Person-specific data, such as age, height, weight, and shoe size were collected at the beginning of the study. Figure 2 illustrates the study design.

### Clinical measures and pre-test assessment

**Figure 3:**
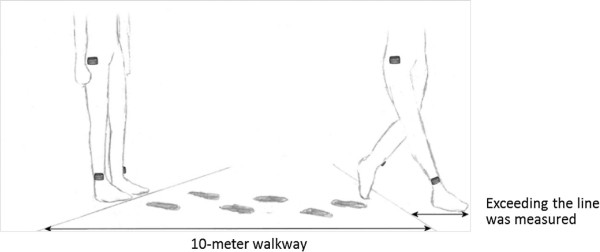
**10-meter walking test.** This figure illustrates the sensors positions during the 10-meter walking test. Number of steps, steps length and the time needed to travel the distance were collected.

The clinical measurements took place in the clinic at the beginning of each phase. These measures were:

1. *10-meter walking test* was used for initial calibration, in which patients were instructed to wear the *move II* (one on the right side hip and two sensors on the right and left ankle) and to walk along a 10 meter flat walkway. Since gait pattern of PwMS differ in various walking speed patients were asked to walk back and forth once at their comfortable walking speed and once again at fastest walking speed. Information about stride length, time and number of steps were recorded by the physician and as raw acceleration data from the *move II* (see Figure 3). This information was used as training data for developing estimation models for both slow and fast walking speed.

2. *Expanded Disability Status Scale EDSS* is defined as a reliable indicator of disability in PwMS [42]. It is an eight functional system scale that includes: motor, sensory, visual, mental and other indicators. The EDSS scale ranges from 0 (no disability) to 10 (death due to MS) in 0.5 unit increments. Patients who score at or less than 5.5 are considered to be able to walk at least 100 m without aid or rest. Patients with score between 6.0 and 8.0 are considered to be ambulatory with limitations. Patients with EDSS score more than 8.0 considered to be totally dependent. Patients’ disease severity and clinical symptoms were assessed using EDSS by an experienced neurologist.

### Ambulatory measures

The activity monitoring system (*move II* activity sensor and pre-configured notebook) was given to the patient at the time of the clinical measurements.

**Figure 4:**
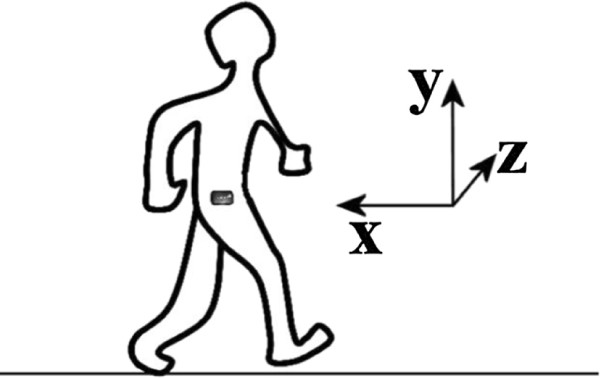
**Sensor position.** This figure illustrates the direction of the three axes and the sensor position during the ambulatory measurement.

**Figure 5:**
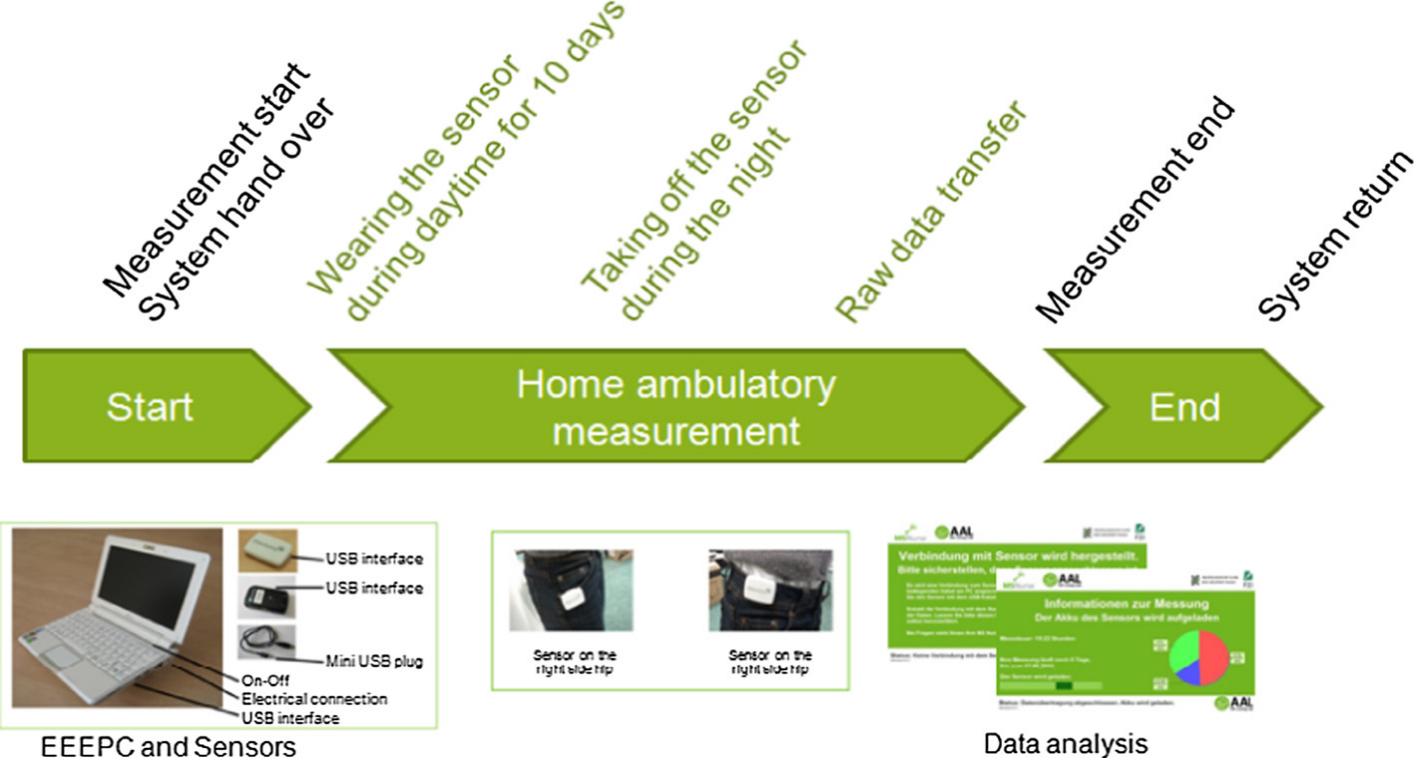
Measurement’s process.

Participants were instructed to carry the *move II* sensor on the right side hip (see Figure 4) up to ten days while carrying out their usual daily activities. They were asked to start carrying the sensor early morning as soon as they get up until they go to bed again (except while swimming, showering and bathing). Furthermore, they were asked to attach it to the notebook via USB before going to sleep. The raw sensor data were transferred automatically and stored on the SD card and the patients got feedback about their daily activity. Therefore, special software for sensor management and physical activity report was developed and installed on the notebook. After ten days, the participants returned the system to the clinic, the data were downloaded to the computer and the participants received a report of their physical activity of the past ten days. Figure 5 illustrates the measurement’s process.

To understand the changes in physical activity characteristics in PwMS, we examined the: a) number of steps calculated for four seconds intervals and then accumulated over the day (number of awake hours) [39], b) mean and maximum walking speed [43], c) physical activity level in terms of MET level (light and moderate to vigorous ModToVig MET level) which is the ratio of the associated metabolic rate for the specific activity divided by the resting metabolic rate (RMR). These values can be obtained from published tables [44]. The specific activity parameters of interest and the operational definitions are shown in Table 1. To assess the impact of the disability on ambulatory activity behaviour, we separated the participants according to their disease severity into two subgroups; mild ambulatory limitation (EDSS = 1 – 2.5) and moderate ambulatory limitation (EDSS = 3 – 5) [45] (Table 2).

**Table 1:** Operational definition of the activity parameters

**Parameter**	**Operational definition**
Number of steps	Average number of steps per time interval (day, week…)
Maximum walking speed	Maximum walking speed over a time interval (day, week…)
Mean walking speed	Average walking speed over a time interval (day, week…)
Physical activity intensity (MET level)	Percentage over a time interval
	(MET = EE / BMR) ; EE: energy expenditure
	BMR: Basal metabolic rate

**Table 2:** Subgroups’ characteristics

		**EDSS 1–2.5**	**EDSS 3 - 5**
		**N = 6**	**N = 5**
**Age**	**Min - Max**	25 - 53	44 - 48
	**Mean (± SD)**	36.14 (±10.53)	46.64 (±1.68)
**Height**	**Min - Max**	158 - 174	164 - 181
	**Mean (± SD)**	165.83 (±6.08)	176 (±6.96)
**Weight**	**Min - Max**	49 - 69	56 - 97
	**Mean (± SD)**	65.33 (±14.04)	79.72 (±17.76)
**EDSS**	**Min - Max**	1 - 2,5	3 - 5
	**Mean (± SD)**	1,75 (±0,82)	4,40 (±0,89)

### Data reduction and statistical analysis

The first day of the ambulatory measurement was not taken into account because the devices were handed out at different hours the first day at the hospital. We considered a valid day of measurement to have ≥ 10 h of wear time. All participants did comply with wearing the sensor 9 days a’ 10 h in all follow-up measurements. The raw data from the accelerometer were imported into MATLAB (R2010a) for offline analysis. The number of steps per measurement as well as the mean walking speed was calculated as an overall average of all days in each measurement. For the activity depended MET level estimation, the activity of the patients was classified and each activity was categorized as light or ModToVig according to [44]. Based on the detected activity class, the energy expenditure was estimated and the MET level value was calculated with the following formula (MET = EE / BMR) [36]. Having both informations (activity class and its corresponding calculated MET value) we defined the MET categories of our patients’ group.

We calculated mean value and standard deviation for each activity parameter. Differences in all activity parameters between two consecutive phases were calculated for each patient. In addition, the differences between the first phase and the follow-up fourth phase for each parameter were assessed non-parametrically by using the Wilcoxon test. To analyse the differences between both subgroups we used the nonparametric Mann–Whitney *U* tests. Wilcoxon test and Mann-Witney *U* tests were used due to the small sample size.

Differences with p ≤ 0.05 were noted as significant. Moreover, we analysed the bivariate correlation between EDSS and the following parameters; number of steps, mean walking speed, max walking speed and MET level. Values between 0.00 and 0.25 was considered as no correlation, values between 0.70 and 0.89 as high correlation and values between 0.90 and 1.00 as very high correlation [46]. Spearman Rho was used for this analysis.

## Results

EDSS was evaluated quarterly and immediately at the beginning of each measurement. While EDSS score did not change throughout the study’s phases in all patients, the physical activity parameters showed differences between each two consecutive phase in both subgroups.

### Patients of mild ambulatory limitation group (EDSS 1–2,5)

**Figure 6:**
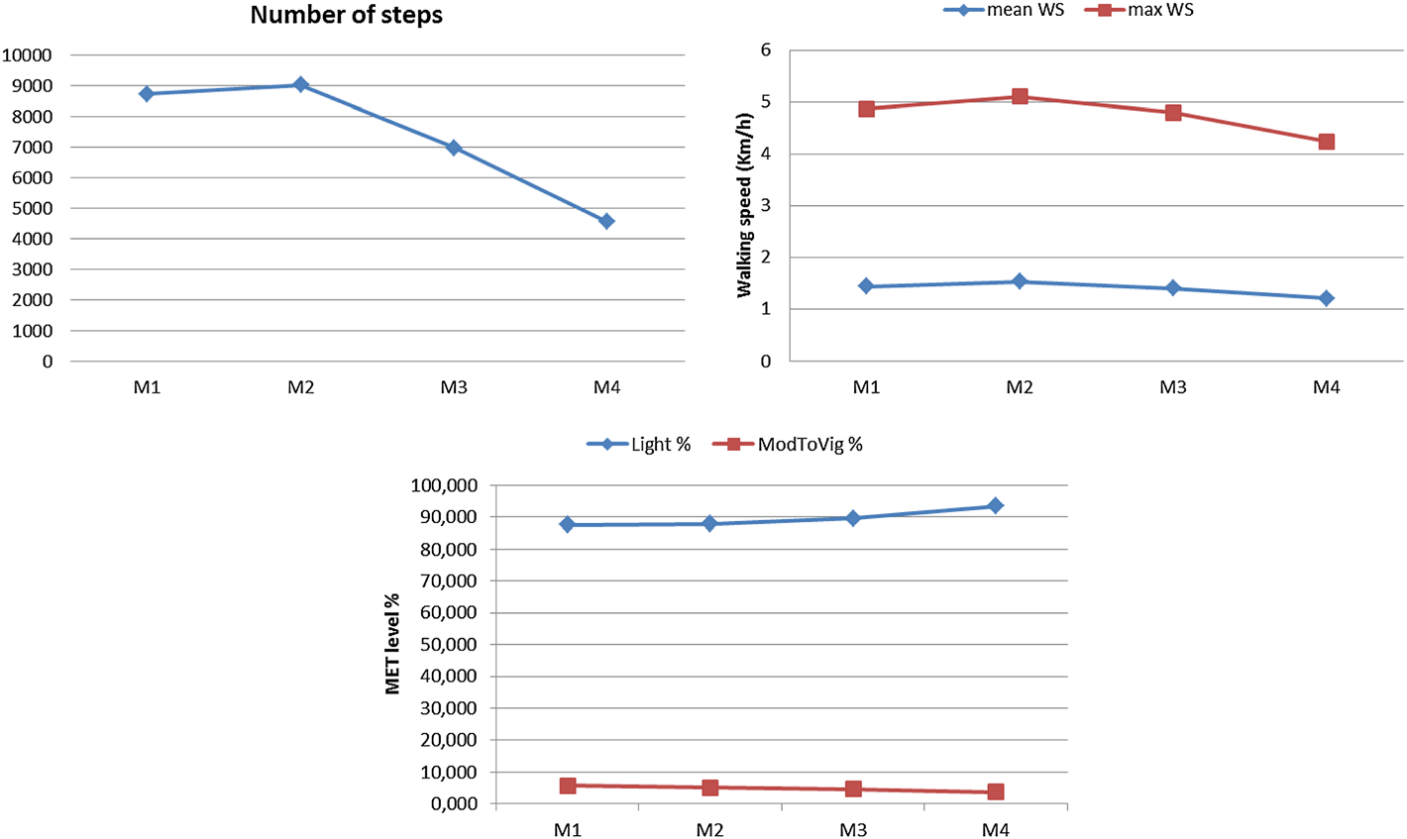
**Changes in physical activity parameters (mild ambulatory limitation).** This figure illustrates an example of the changes in physical activity parameters over the study time in the patients of the mild ambulatory limitation subgroup. An increment was noticed in daily number of steps between the first and the second measurement. This increment was combined with slightly increase in walking speed. In comparison to baseline, patient showed decline in all activity parameters in the follow-up fourth measurement.

Patients in this group showed increment in the number of steps (~1091 steps) between the first phase and the follow-up second phase. Only one patient showed decline between these two phases (~292). Remarkably, these changes in number of steps were combined with slightly increases in mean and max walking speed in 4 patients of this group (1.8%, 1.13%), respectively. One patient did not show any change neither in mean nor in maximum walking speed. In average, participants in mild limitation group showed increases in number of steps, mean walking speed and maximum walking speed and in ModToVig MET level between first phase and the follow-up second phase. However, they all showed a decline in all parameters between the second and the third phase as well as between the third and the fourth phase. In comparison to baseline, they showed decline in steps (~ 1683 steps), mean walking speed and max walking speed (-0.12 Km/h, -0.16 Km/h), respectively. ModToVig MET level did not show significant change (-0.9%) between the first and the follow-up fourth measurement. Figure 6 illustrates an example of these changes in one patient of the mild ambulatory limitation group.

### Patients of moderate ambulatory limitation group (EDSS 3–5)

**Figure 7:**
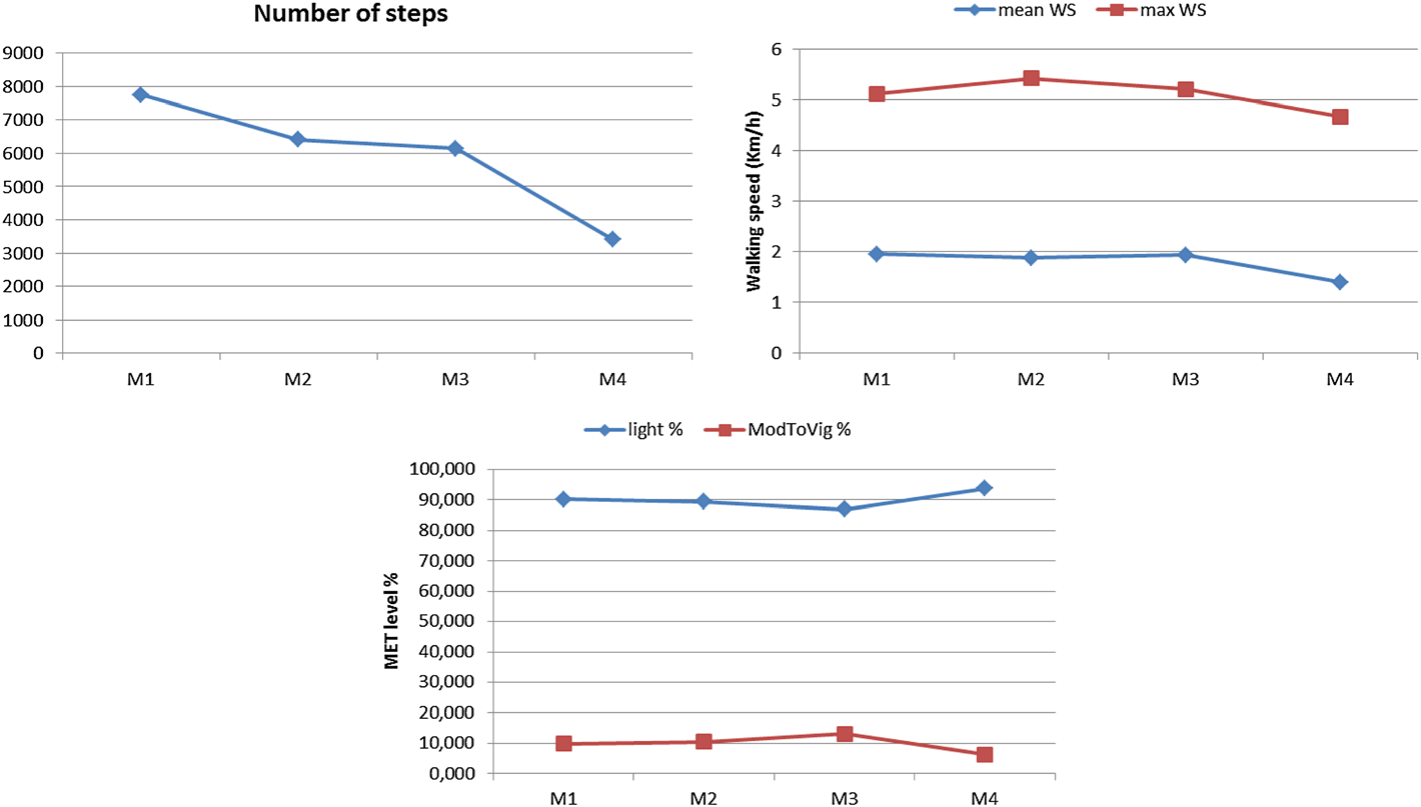
**Changes in physical activity parameters (moderate ambulatory limitation).** This figure illustrates an example of the changes in physical activity parameters over the study time in the patients of the moderate ambulatory limitation subgroup. In comparison to baseline, patient showed decline in all activity parameters in the follow-up fourth measurement.

Patients of this group showed a decline in all physical activity parameter between each two consecutive phases. Only one patient showed an increase in daily number of steps (~900) between the second and the follow-up third phase. Furthermore, a slightly increase in maximum walking speed between the first measurement and the follow-up second one was noticed in another patient. However, this patient did not show any increment in daily number of steps or ModToVig MET level. In comparison to the baseline, all patients showed decline in daily number of steps (~1673), a slight decline in mean and max walking speed (8.7%, 2.6%), respectively, and in ModToVig MET level (-1.4%) between first and follow-up fourth measurement. An example of the decline in different activity parameters is shown in Figure 7.

### All patients combined measurements

In comparison to baseline, 81% of the participant showed a significant decline in the number of steps (-23.20%, p = 0.008) as well as in light and ModToVig MET level (-14.5%, p = 0.03), 63% showed reduction in maximum walking speed (-5.3%, p = 0.02) between first phase and follow-up fourth. Mean walking speed did not demonstrate a significant decline (1.7%, p = 0.75). The mean daily number of steps over all measurements was 7164.93 ± 2950.98. This value is consistent to the value reported in [47], and comparable to the average number of steps reported in [30]. Our value is more than the values reported in [48,49]. Mean walking speed ranged from 0.55 to 1.96 km/h (mean = 1.29 ± 0.37); maximum walking speed ranged from 2.31 to 5.87 km/h (mean = 4.49 ± 0.98). Percentage of day spent in light activity level ranged from 86.21 to 99.22 (mean = 90.23 ± 3.63).

### Between groups differences

**Figure 8:**
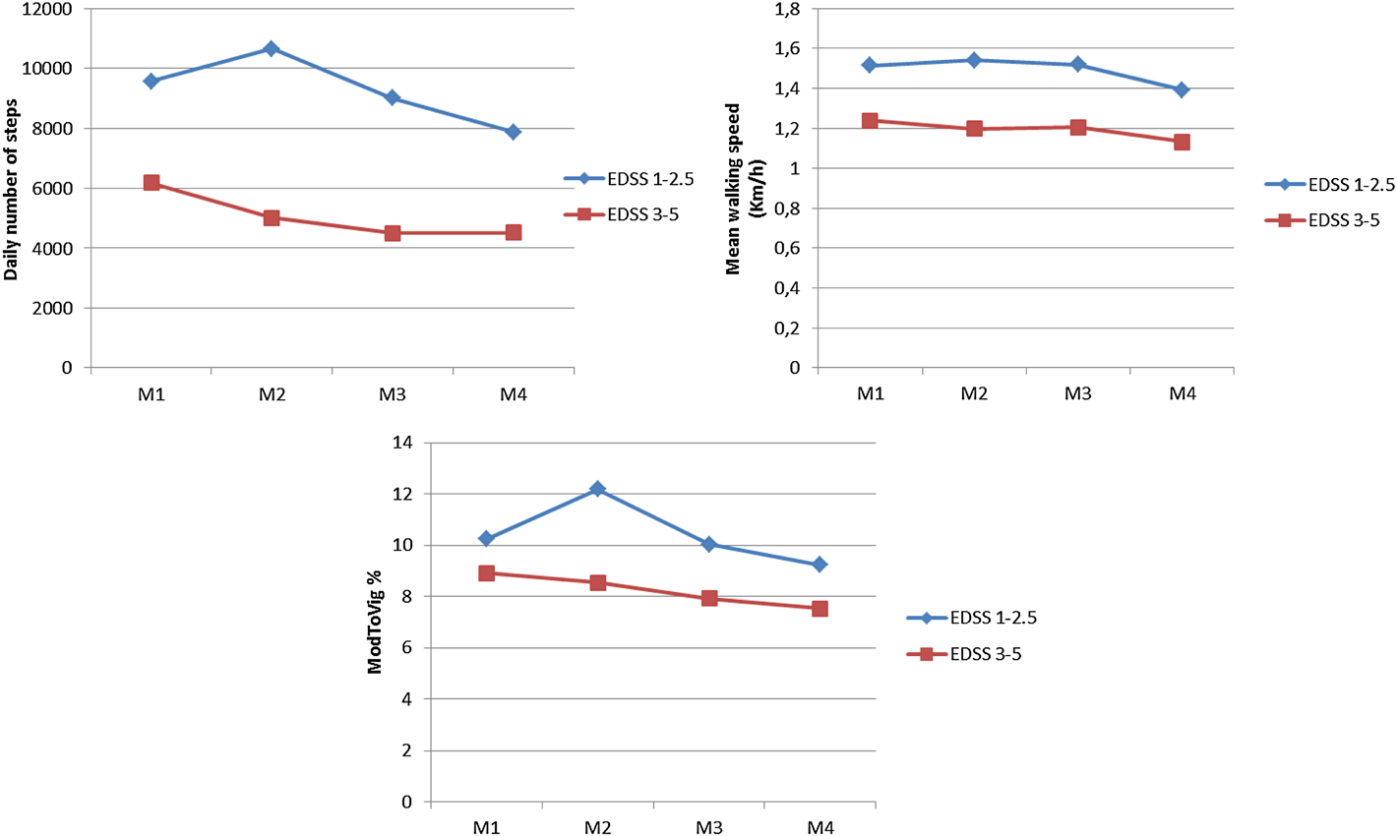
**Differences between both subgroups.** This figure illustrates the differences between both subgroups. Subgroup (EDSS 1–2.5) accumulated significantly more number of steps (p < 0.005) than the subgroup (EDSS 3–5), and they had faster mean walking speed (p = 0.03). Differences in ModToVig MET level were not significant between both subgroups (p = 0.1).

In average over all measurements the mild affected MS subgroup accumulated significantly more steps and had faster mean walking speed compared to moderate affected MS subgroup (9214.33 ± 2439.11 vs. 5018,13 ± 2416.96, p < 0.005; 1.48 ± 0.19 vs. 1.12 ± 0.44, p = 0.03), respectively. Furthermore, a marginal difference between the subgroups in maximum walking speed was noticed (5.02 ± 0.5 vs. 3.84 ± 0.97, p = 0.08). On the contrary, light MET level as well as ModToVig MET level demonstrated non-significant differences between both MS subgroups (88.41% ± 4.24% vs. 92.31% 4.47%, p = 0.1; 11.87% ± 2.8% vs. 7.79% ± 4.46%; p = 0.1), respectively (see Figure 8 and Table 3).

**Table 3:** Differences between subgroups

	**EDSS 1–2.5**		**EDSS 3 - 5**		**p-value**
	**Mean ± SD**		**Mean ± SD**		
**Step counts**	9214.33	±2439.11	5018.13	±2416.96	< 0.005
**Maximum walking speed**	5.02	±0.5	3.84	±0.97	0.08
**Mean walking speed**	1.48	±0.19	1.12	±0.44	< 0.05
**MET level (Light%)**	88.41	±4.24	92.31	±4.47	0.1
**MET level (ModToVig%)**	11.87	±2.8	7.79	±4.46	0.1

Relatively weak correlation was found between the total number of steps and the EDSS score (r = -0.54, p = 0.08). High inverse correlation was noticed between mean walking speed and EDSS score (r = -0.71, p = 0.01), whereas maximum walking speed did not show a significant correlation with EDSS (r = -0.37, p = 0.2). Both light and ModToVig MET levels were not significantly correlated with r = 0.36; p = 0.2 and r = -0.36; p = 0.2), respectively. The correlation coefficients for ambulatory activity parameters and neurological parameters (EDSS) are shown in Table 4.

**Table 4:** Bivariate relationships between ambulatory activity parameters and EDSS

	**Number of steps**	**Maximum walking speed**	**Mean walking speed**	**(Light%)**	**(ModToVig%)**	**EDSS**
**Number of steps**	1	0.727^*^	0.758^*^	-0.636^**^	0.636^**^	-0.541^**^
**Maximum walking speed**		1	0.782^*^	-0.327	0.327	-0.376
**Mean walking speed**			1	-0.155	0.155	-0.706^*^
**(Light%)**				1	-1	0.358
**(ModToVig%)**					1	-0.358
**EDSS**						1

*(p ≤ .01), **(p ≤ .05).

## Discussion

The results of this pilot study showed that: 1) a simple tri-axial accelerometer sensor adapted for long-term monitoring can be used to capture the changes in ambulatory physical activity parameters of PwMS objectively; 2) these parameters are more responsive to slight disability changes than the clinical measures. As hypothesised, the PwMS demonstrated significant decline in ambulatory activity at follow-up, which was revealed by a lower number of steps, reduced ModToVig MET level and slower maximum walking speed. In addition, we found that patients with mild ambulatory limitation accumulate more daily steps than moderate ambulatory limitation patients. Indeed, there was a large different in daily number of steps accumulated with patients of mild ambulatory limitation and moderate ambulatory limitation (~4000 steps). This value is comparable with the difference in daily number of steps reported in [50]. We have also observed that patients in the mild ambulatory limitation subgroup could have some improvement in the physical activity behaviour. Moderate ambulatory limitation walked significantly slower than patients with light ambulatory limitation. As expected, walking speed and number of steps are negatively correlated to the EDSS score (r = -0.71, r = -0.54), respectively. The association was also reported in [51]. This observed correlation is important and provides evidence that physical activity parameters measured by the accelerometer can be used to monitor the patient’s clinical status.

Our findings regarding the number of steps accumulated per day are comparable with other studies [52]. For example, average of daily number of steps reported in the study of [31] was (steps/day = 7698), whereas the participants in our study accumulated an average of approx. 7000 steps per day. Patients of moderate disability tented to be less active as it is reported in [50,52,53], and this result is in line with our findings. Furthermore, consistent with previous reports [54,55], participants showed attenuated walking speed. Moreover, mean walking speed showed negative correlation with the neurological parameter (EDSS), which fits the findings in [54].

Contrary to our hypothesis, no significant differences between both subgroups were noticed in physical activity intensity (MET level). Moreover, the MET level showed non-significant correlation with EDSS which is not in line with previous research [35].

Our findings indicate that accelerometer can capture and reflect the changes in physical activity and walking ability among ambulatory PwMS in real-life environment. These results are in line with [34] and suggest that the accelerometer’s outcome can be used as early signs of changes in disability, which is not reflected by the EDSS score and could be considered as a clinical tool for evaluating interventions in multiple sclerosis.

The idea of using accelerometers to capture physical activity behaviour in patients has been investigated in different studies and in the context of different diseases, not limited to MS. Some of these studies used pedometer-based systems that provide information about the number of steps accumulated by the patients. Nevertheless, such systems do neither provide information about walking quality or speed nor about activity intensity [56,57]. Other studies have investigated the use of 3D accelerometers but either in clinical environment with short discrete motor tasks or over short periods (e.g. 7-days) [35,58]. To our knowledge, this is the first study that used a tri-axial accelerometer home-based system to monitor the ambulatory physical activity and walking impairment parameter in PwMS objectively over a longer period of time with multiple phases. However, further investigations are needed to determine which factor can lead to meaningful clinical improvement. Such research might allow for better design of therapeutic and medical interventions based on accelerometry. Furthermore, our observation and findings shed a light on using accelerometer as a tool to determine the benefit of the therapy or medication not only in PwMS but in other chronic disease with physical activity limitation such as; Parkinson’s disease, Amyotrophic lateral sclerosis (ALS) and stroke.

A limitation of this pilot study is the small sample size. Furthermore, seasonality may have contributed to the reduction in physical activity parameter since our data were collected between May and February which means that data collection covered winter time when physical activity is at least lowest [59]. Nevertheless, PwMS may also exhibit temporary worse symptoms when the weather is warm [60]. However, this external factor was not considered in this study. Additionally, the effect of the disease’s duration on the individual physical activity behaviour should be analysed in future works. Furthermore, we cannot generalise our results to all the MS population because the analysis was limited to patients with ambulatory ability without assistance. Using the notebook for data collection and feedback system is considered as major limitation in large scale study and it imposes an obstacle to real-time data access and analysis. In order to overcome this limitation we aim to develop a cloud-dependent system for data management and analysis. The data will be available for both medical staff and patients throughout special mobile web-based applications. The doctor’s software application enables clinical professionals to supervise patients in their real-world environment, so they can make wiser decisions. The patient’s software allows patients to monitor their daily activity and get feedback from the physicians.

## Conclusions

Changes in ambulatory physical parameters (number of steps, mean and maximum walking speed and physical activity intensity) of PwMS were recorded using an activity sensor over one year. Our results suggest that the parameters extracted from one tri-axial accelerometer can be used as an objective measurement system that provides insights into the physical activity behaviour of PwMS in their every-day living environment. Moreover, we suggest that this information about the changes in physical activity using home-based ambulatory monitoring system may help to develop appropriate treatment interventions for PwMS.

## Competing interests

The authors declare that no competing interests exist.

## Authors’ contributions

LS participated in the study concept and design, took responsibility for the data processing and analysis and drafted the manuscript. TZ participated in the design and coordination of the study, was responsible for the technical and material support and revised the manuscript. BvH contributed in the statistical data analysis and interpretation. SS was responsible for the acquisition of the data and revised the manuscript. SH contributed in the coordination of the study and revised the manuscript. AR conceived the study, participated in the design and coordination of the study, contributed in the data analysis and presentation, critically revised the manuscript. All authors read and approved the final manuscript.

## References

[B1] NG AV Kent-Braun JA Quantification of lower physical activity in person with multiple sclerosis Med Sci Sports Exerc 1997 29 517 523 9107635 10.1097/00005768-199704000-00014

[B2] Dallmeijer AJ Beckerman H De GV Van de Port IG Lankhorst GJ Dekker J Long-term effect of comorbidity on the course of physical functioning in patients after stroke and with multiple sclerosis J Rehabil Med 2009 41 322 326 10.2340/16501977-0335 19363563

[B3] Statement ATS Guidelines for the Six-Minute Walk Test Am J Respir Crit Care Med 2002 166 111 117 12091180 10.1164/ajrccm.166.1.at1102

[B4] Fischer JS Jak AJ Kniker JE Rudick RA Cutter G Guidebook - MSFC Manual and Forms. Administration and Scoring Manual 2001

[B5] Motl R Snook E Schapiro R Symptoms and physical activity behavior in individuals with multiple sclerosis Res Nurs Health 2008 31 466 475 10.1002/nur.20274 18286635

[B6] Polman C Reingold S Banwell B Clanet M Cohen J Filippi M Fujihara K Havrdova E Hutchinson M Kappos L Lublin F Montalban X O’Connor P Sandberg-Wollheim M Thompson A Waubant E Weinshenker B Wolinsky J Diagnostic criteria for multiple sclerosis: 2010 Revisions to the McDonald criteria Ann Neurol 2011 69 292 302 10.1002/ana.22366 21387374 PMC3084507

[B7] Bobholz JA Rao SM Cognitive dysfunction in multiple sclerosis: a review of recent developments Curr Opin Neurol 2003 16 283 288 10.1097/00019052-200306000-00006 12858063

[B8] Macko RF Haeuber E Shaughnessy M Coleman KL Boone DA Smith GV Silver KH Microprocessor-based ambulatory activity monitoring in stroke patients Med Sci Sports Exerc 2002 34 394 399 11880800 10.1097/00005768-200203000-00002

[B9] Prince SA Adamo KB Hamel M Hardt J Connor GS Tremblay M A comparison of direct versus self-report measures for assessing physical activity in adults: a systematic review Int J Behav Nutr Phys Act 2008 5 56 10.1186/1479-5868-5-56 18990237 PMC2588639

[B10] Harris TJ Owen CG Victor CR Adams R Ekelund UL Cook DG A Comparison of Questionnaire, Accelerometer, and Pedometer Med Sci Sports Exerc 2009 41 1392 1402 19516162 10.1249/MSS.0b013e31819b3533

[B11] Motl RW Zhu W Park Y McAuley E Scott JA Snook EM Reliability of scores from physical activity monitors in adults with multiple sclerosis Adapt Phys Activ Q 2007 24 245 253 17916920 10.1123/apaq.24.3.245

[B12] Bussmann JBJ Martens WLJ Tulen JHM Schasfoort FC Berg-Emons HJG Stam HJ Measuring daily behavior using ambulatory accelerometry: The Activity Monitor Behav Res Methods Instrum Comput 2001 33 349 356 10.3758/BF03195388 11591066

[B13] Speelman AD Van Nimwegen M Borm GF Bloem BR Munneke M Monitoring of walking in Parkinson’s disease: Validation of an ambulatory activity monitor Parkinsonism Relat Disord 2011 17 402 404 10.1016/j.parkreldis.2011.02.006 21367643

[B14] White DK Wagenaar RC Del Olmo ME Ellis TD Test-retest reliability of 24 hours of activity monitoring in individuals with Parkinson’s disease in home and community Neurorehabil Neural Repair 2007 21 327 340 10.1177/1545968306297867 17369513

[B15] Salarian A Russmann H Vingerhoets F Burkhard PR Aminian K Ambulatory Monitoring of Physical Activities in Patients With Parkinson’s Disease IEEE Trans Biomed Eng 2007 54 2296 2299 18075046 10.1109/tbme.2007.896591

[B16] Busse ME Pearson OR Van Deursen R Wiles CM Quantified measurement of activity provides insight into motor function and recovery in neurological disease J Neurol Neurosurg Psychiatry 2004 75 884 888 10.1136/jnnp.2003.020180 15146006 PMC1739073

[B17] Motl RW McAuley E Snook EM Scott JA Accuracy of two electronic pedometers for measuring steps taken under controlled conditions among ambulatory individuals with multiple sclerosis Mult Scler 2005 11 343 345 10.1191/1352458505ms1161oa 15957518

[B18] Rietberg MB Van W Erwin E Uitdehaag BM De V Henrica C Kwakkel G How Reproducible Is Home-Based 24-Hour Ambulatory Monitoring of Motor Activity in Patients With Multiple Sclerosis? Arch Phys Med Rehabil 2010 91 1537 1541 10.1016/j.apmr.2010.07.018 20875511

[B19] Kinnunen TI Tennant PWG McParlin C Poston L Robson SC Bell R Agreement between pedometer and accelerometer in measuring physical activity in overweight and obese pregnant women BMC Public Health 2011 11 501 10.1186/1471-2458-11-501 21703033 PMC3141462

[B20] Motl RW Sandroff BM Objective monitoring of physical activity behavior in multiple sclerosis Phys Ther Rev 2010 15 204 211 10.1179/174328810X12814016178953

[B21] Najafi B Vaziri A Boloori AR Ambulatory system for measuring and monitoring physical activity and risk of falling and for automatic fall detection 2012

[B22] Luinge H Veltink PH Inclination Measurement of Human Movement Using a 3-D Accelerometer With Autocalibration IEEE Trans. Neural Syst. Rehabil. Eng 2004 12 112 121 10.1109/TNSRE.2003.822759 15068194

[B23] Plasqui G Westerterp KR Physical activity assessment with accelerometers: an evaluation against doubly labeled water Obesity (Silver Spring) 2007 15 2371 2379 10.1038/oby.2007.281 17925461

[B24] Vanhelst J Theunynck D Gottrand F Béghin L Reliability of the RT3 accelerometer for measurement of physical activity in adolescents J Sports Sci 2010 28 375 379 10.1080/02640410903502790 20175016

[B25] Motl RW McAuley E Snook EM Scott JA Validity of physical activity measures in ambulatory individuals with multiple sclerosis Disabil Rehabil 2006 28 1151 1156 10.1080/09638280600551476 16966236

[B26] Klassen L Schachter C Scudds R An exploratory study of two measures of free-living physical activity for people with multiple sclerosis Clin Rehabil 2007 22 260 271 10.1177/0269215507082740 18285434

[B27] Motl RW McAuley E Association between change in physical activity and short-term disability progression in multiple sclerosis J Rehabil Med 2011 43 305 310 10.2340/16501977-0782 21305247

[B28] Motl R Goldman Benedict B Walking impairment in patients with multiple sclerosis: exercise training as a treatment option Neuropsychiatr Dis Treat 2010 6 767 774 21173883 10.2147/NDT.S10480 PMC2999522

[B29] Iezzoni LI Rao SR Kinkel RP Experiences Acquiring and Using Mobility Aids Among Working-Age Persons with Multiple Sclerosis Living in Communities in the United States Am J Phys Med Rehabil 2010 89 1010 1023 10.1097/PHM.0b013e3181f70292 20881588

[B30] Sosnoff JJ Weikert M Dlugonski D Smith DC Motl RW Quantifying gait impairment in multiple sclerosis using GAITRite™ technology Gait Posture 2011 34 145 147 10.1016/j.gaitpost.2011.03.020 21531562

[B31] Weikert M Suh Y Lane A Sandroff B Dlugonski D Fernhall B Motl RW Accelerometry is associated with walking mobility, not physical activity, in persons with multiple sclerosis Med Eng Phys 2012 34 590 597 10.1016/j.medengphy.2011.09.005 21968005

[B32] Sosnoff JJ Goldman MD Motl RW Real-life walking impairment in multiple sclerosis: preliminary comparison of four methods for processing accelerometry data Mult Scler 2010 16 868 877 10.1177/1352458510373111 20534642

[B33] Hobart JC Riazi A Lamping DL Fitzpatrick R Thompson AJ Measuring the impact of MS on walking ability: the 12-Item MS Walking Scale (MSWS-12) Neurology 2003 60 31 36 10.1212/WNL.60.1.31 12525714

[B34] Motl RW Dlugonski D Suh Y Weikert M Fernhall B Goldman M Accelerometry and its association with objective markers of walking limitations in ambulatory adults with multiple sclerosis Arch Phys Med Rehabil 2010 91 1942 1947 10.1016/j.apmr.2010.08.011 21112438 PMC3165019

[B35] Motl RW Sosnoff JJ Dlugonski D Suh Y Goldman M Does a waist-worn accelerometer capture intra- and inter-person variation in walking behavior among persons with multiple sclerosis? Med Eng Phys 2010 32 1224 1228 10.1016/j.medengphy.2010.08.015 20875952 PMC3165016

[B36] von Haaren B Anastasopoulou P Haertel S Hey S Commonly used single regression model compared to activity based method to predict energy expenditure Poster presented at 3rd International Conference on Ambulatory Monitoring of Physical Activity and Movement in Amherst (MA), USA 2013

[B37] Motl RW McAuley E Sandroff BM Longitudinal Change in Physical Activity and Its Correlates in Relapsing-Remitting Multiple Sclerosis Phys Ther 2013 93 1037 1048 10.2522/ptj.20120479 23599354

[B38] Anastasopoulou P Härtel S Hey S A comparison of two commercial activity monitors for measuring step counts during different everyday life walking activities Int J Sports Med Sci Eng 2013 7 031 035

[B39] Anastasopoulou P Tansella M Stumpp J Shammas L Hey S Classification of human physical activity and energy expenditure estimation by accelerometry and barometry 2012 34th Annual International Conference of the IEEE Engineering in Medicine and Biology Society (EMBC) 2012 6451 6454 10.1109/EMBC.2012.6347471 23367406

[B40] Härtel S Gnam J Löffler S Bös K Estimation of energy expenditure using accelerometers and activity-based energy models—validation of a new device Eur Rev Aging Phys Act 2011 8 109 114 10.1007/s11556-010-0074-5

[B41] Kurtzke JF Rating neurologic impairment in multiple sclerosis: an expanded disability status scale (EDSS) Neurology 1983 33 1444 1452 10.1212/WNL.33.11.1444 6685237

[B42] Goodkin DE Cookfair D Wende K Bourdette D Pullicino P Scherokman B Whitham R Inter- and intrarater scoring agreement using grades 1.0 to 3.5 of the Kurtzke Expanded Disability Status Scale (EDSS). Multiple Sclerosis Collaborative Research Group Neurology 1992 42 859 863 10.1212/WNL.42.4.859 1565242

[B43] Anastasopoulou P Shammas L Hey S Assessment of Human Gait Speed and Energy Expenditure Using a Single Triaxial Accelerometer 2012

[B44] Ainsworth BE Haskell WL Herrmann SD Meckes N Bassett DR Tudor-locke C Greer JL Vezina J Whitt-glover MC Leon AS 2011 Compendium of Physical Activities Med Sci Sports Exerc 2011 43 1575 1581 21681120 10.1249/MSS.0b013e31821ece12

[B45] Goldman MD Marrie RA Cohen JA Evaluation of the six-minute walk in multiple sclerosis subjects and healthy controls Mult Scler 2008 14 383 390 10.1177/1352458507082607 17942508

[B46] Bortz J Statistik für Human- und Sozialwissenschaftler 2005 6 Berlin, Heidelberg, New York: Springer

[B47] Sandroff BM Dlugonski D Weikert M Suh Y Balantrapu S Motl RW Physical activity and multiple sclerosis: new insights regarding inactivity Acta Neurol Scand 2012 126 256 262 22211941 10.1111/j.1600-0404.2011.01634.x

[B48] Dlugonski D Pilutti LA Sandroff BM Suh Y Balantrapu S Motl RW Steps Per Day Among Persons With Multiple Sclerosis: Variation by Demographic, Clinical, and Device Characteristics Arch Phys Med Rehabil 2013 94 1534 1539 10.1016/j.apmr.2012.12.014 23419331

[B49] Gosney JL Scott JA Snook EM Motl RW Physical activity and multiple sclerosis: validity of self-report and objective measures Fam Community Health 2007 30 144 150 10.1097/01.FCH.0000264411.20766.0c 19241650

[B50] Romberg A Ruutiainen J Daumer M Physical Activity in Finnish Persons with Multiple Sclerosis J Nov Physiother 2013 3 150 155

[B51] Nogueira Leandro Alberto C Dos Santos Luciano T Sabino PG Alvarenga Regina Maria P Santos T Luiz C Factors for Lower Walking Speed in Persons with Multiple Sclerosis Mult Scler Int 2013 2013 1 8 10.1155/2013/875648 PMC3628672 23606966

[B52] Cavanaugh JT Gappmaier VO Dibble LE Gappmaier E Ambulatory Activity in Individuals With Multiple Sclerosis J Neurol Phys Ther 2011 35 26 33 10.1097/NPT.0b013e3182097190 21475081

[B53] Snook EM Motl RW Gliottoni RC The effect of walking mobility on the measurement of physical activity using accelerometry in multiple sclerosis Clin Rehabil 2009 23 248 258 10.1177/0269215508101757 19218299

[B54] Givon U Zeilig G Achiron A Gait analysis in multiple sclerosis: Characterization of temporal–spatial parameters using GAITRite functional ambulation system Gait Posture 2009 29 138 142 10.1016/j.gaitpost.2008.07.011 18951800

[B55] Burschka JM Keune PM Menge U Oy U Oschmann P Hoos O An exploration of impaired walking dynamics and fatigue in Multiple Sclerosis BMC Neurol 2012 12 161 10.1186/1471-2377-12-161 23270547 PMC3547727

[B56] Prajapati SK Gage WH Brooks D Black SE McIlroy WE A Novel Approach to Ambulatory Monitoring: Investigation Into the Quantity and Control of Everyday Walking in Patients With Subacute Stroke Neurorehabil Neural Repair 2010 25 6 14 20829413 10.1177/1545968310374189

[B57] Dobkin BH Xu X Batalin M Thomas S Kaiser W Reliability and Validity of Bilateral Ankle Accelerometer Algorithms for Activity Recognition and Walking Speed After Stroke Stroke 2011 42 2246 2250 10.1161/STROKEAHA.110.611095 21636815 PMC4337400

[B58] Kos D Nagels G D’Hooghe MB Duquet W Ilsbroukx S Delbeke S Kerckhofs E Measuring Activity Patterns Using Actigraphy in Multiple Sclerosis Chronobiol Int 2007 24 345 356 10.1080/07420520701282364 17453852

[B59] Pivarnik JM Reeves MJ Rafferty AP Seasonal variation in adult leisure-time physical activity Med Sci Sports Exerc 2003 35 1004 1008 12783049 10.1249/01.MSS.0000069747.55950.B1

[B60] Heesen C Romberg A Gold S Schulz K Physical exercise in multiple sclerosis: supportive care or a putative disease-modifying treatment Expert Rev Neurother 2006 6 347 355 10.1586/14737175.6.3.347 16533139

